# Gut Microbiota and Immunity: Possible Role in Sudden Infant Death Syndrome

**DOI:** 10.3389/fimmu.2015.00269

**Published:** 2015-06-03

**Authors:** Paul N. Goldwater

**Affiliations:** ^1^Discipline of Paediatrics, School of Paediatrics and Reproductive Health, University of Adelaide, North Adelaide, SA, Australia

**Keywords:** sudden infant death syndrome, gut, microbiome, immunity

## Abstract

The gut microbiome influences the development of the immune system of young mammals; the establishment of a normal gut microbiome is thought to be important for the health of the infant during its early development. As the role of bacteria in the causation of sudden infant death syndrome (SIDS) is backed by strong evidence, the balance between host immunity and potential bacterial pathogens is likely to be pivotal. Bacterial colonization of the infant colon is influenced by age, mode of delivery, diet, environment, and antibiotic exposure. The gut microbiome influences several systems including gut integrity and development of the immune system; therefore, gut microflora could be important in protection against bacteria and/or their toxins identified in SIDS infants. The aims of the review are to explore (1) the role of the gut microbiome in relation to the developmentally critical period in which most SIDS cases occur; (2) the mechanisms by which the gut microbiome might induce inflammation resulting in transit of bacteria from the lumen into the bloodstream; and (3) assessment of the clinical, physiological, pathological, and microbiological evidence for bacteremia leading to the final events in SIDS pathogenesis.

## Introduction

The common bacterial hypothesis (1, 2) and the role of bacteria in the causation of sudden infant death syndrome (SIDS) have not been considered in depth by mainstream researchers nor has there been broad interest in the potential contribution of infection and inflammation to these deaths. Studies on the microbiology of SIDS (3–7) provided explanations for SIDS risk factors and potential mechanisms in which inflammatory responses could affect abnormal arousal, respiration (8, 9), and/or brain stem compromise (10); these are areas that have preoccupied the mainstream of SIDS research for decades without successfully providing hypotheses congruent with epidemiological and pathological features of SIDS.

The infection model of SIDS stands on substantial evidence identifying: *Staphylococcus aureus* and its enterotoxins (11–14); toxigenic *Escherichia coli* (15, 16); *Clostridium perfringens* (3, 4); and recent findings (based on a restricted number of bacterial species identified by culture and PCR) of significant differences in the gut microbiome between SIDS and healthy babies (17). A review of the gut microbiome of babies in the context of immunity and immune/inflammatory responses to bacterial infection was considered timely: to help clarify the role of gut/mucosal immunity in relation to SIDS; to explain the apparent mucosal dysregulation reported for one SIDS infant (18). The basis for a link between the gut microbiome and SIDS is founded upon published evidence that provides a logical explanation of underlying pathomechanisms involved in SIDS. The evidence is summarized in Table 1 and addressed later in the review.

**Table 1:** **Comparison between findings in SIDS compared to sepsis**.

	SIDS	Sepsis
Pathological findings	Vasculopathy	Vasculopathy
	(intra-thoracic petechial hemorrhages)	
	Coagulopathy	Coagulopathy
	(raised FDPs)	(raised FDPs)
	Heavy, wet, congested lungs	Heavy, wet, congested lungs
	Renal shutdown	Renal shutdown
	(empty bladder)
	Evidence of recent pro-inflammatory cytokine release	Evidence of recent pro-inflammatory cytokine release
	Cerebro-spinal microgliosis	?Cerebro-spinal microgliosis
	Vasculopathy	Vasculopathy
	Coagulopathy	Coagulopathy
	Raised CSF IL-6	
	Raised rectal temperature (fever)	Raised temperature (fever)
Clinical findings	Sweatiness (fever)	Fever
	Recent gastrointestinal or respiratory viral infection	Underlying infection
Physiological findings	Hypoxemia, tachycardia then bradycardia, asystole, gasping, death	Hypoxemia, tachycardia then bradycardia, asystole, gasping, death
	Raised rectal temperature	Fever
Microbiological findings	Normally sterile site infection	Normally sterile site infection
	Evidence of bacteremia	Bacteremia
Risk factors	Genetic (various; immune gene polymorphisms)	Genetic (various)
	Prenatal (exposure to smoke products)	Prenatal (various)
	Postnatal (exposure to smoke products, prone sleep, etc.)	Postnatal (smoking, immunopathy)

*?, undetermined*.

## Background

It has been known since 1905 that the microbiota of neonates undergo change with growth and development (19). The type of early microbiota is important in terms of development of the immune system, and this could influence susceptibility to infection, induction of gut inflammation, and adverse outcomes of infection. Improved understanding of how the gut microbiota and type of feeding affect immune and neurodevelopment has arisen since the introduction of molecular techniques to identify and quantify bacterial genera and species involved (17, 20–22).

A literature search reviewed recent publications covering development of the infant gut microbiota in conjunction with development of the infant immune system and related functions. Articles pertaining to the gut microbiota and SIDS/sudden unexplained infant deaths (SUDI) were also reviewed. Search engines used included PubMed, MedlineRanker, PubCrawler, Google Scholar, and Open Access Library (OALib).

## Role of Gut Microbiome during the Critical Developmental Period Associated with SIDS

### Colonization

Bacterial colonization of the human infant colon is influenced by many factors – age, mode of delivery (23), diet, environment, and antibiotic exposure (20, 21, 24–26).

There is growing evidence that cesarean section (CS) (not regarded as a SIDS risk factor when controlled for gestational age) (27) is linked to an impoverishment of natural development of the immune system. Exposure of the neonatal gut to bacterial priming (as occurs with vaginal delivery) appears to be missed in babies born via CS (28). One of the main associations with CS is low gestational age. Prematurity remains a significant and largely unexplained risk factor for SIDS; the vulnerable, immature host notwithstanding (29). It is presumed that the abovementioned mechanisms (27) over and above the vulnerable host would go some way to explain this increased risk.

Diet influences the gut microbiota. The effect of breast milk on the infant immune system is considered to derive benefit via its effect on gut bacterial colonization (30). Breast feeding has a protective effect against SIDS and given the effects on the gut microbiota, suggests that these bacteria might play a role in addition to maternally transferred cellular and humoral immunity. CS remains the most common mode of delivery of preterm infants. The possibility that these babies will receive breast milk or enteral feeds remains remote while the opposite is true for receiving treatment with antibiotics. Animal studies show that a lack of enteral nutrition may be associated with an increased risk of septic shock due to bacterial translocation caused by intestinal epithelial cell apoptosis (31). These factors thus adversely affect preterm infants in terms of their susceptibility to infection and to inflammatory gut disease such as necrotizing enterocolitis (NEC) (30). Because SIDS infants are more likely to have had symptoms of infectious diseases in the last week or the last day before death, they are more often examined by a physician and given antibiotics than control babies (32). It remains unknown if antibiotics specifically contribute to the risk of SIDS.

### Gut immunology and homeostasis

The intestinal immune system is modulated in response to environmental factors shortly after birth (33). Battersby and Gibbons (34) summarized the emerging knowledge of how the gut maintains immune homeostasis with non-pathogenic bacteria (34). A better understanding of the molecular and cellular mechanisms sustaining homeostasis is emerging (35–37).

## Mechanisms by Which the Gut Microbiome Could Induce Inflammation and Transit of Bacteria to the Blood Stream

### Microbial recognition and inflammation

The nuclear factor kappa B (NF-κB) pathway is responsible for microbial recognition and inflammatory responses in the adult gut. NF-κB, the so-called “master switch” of the immune system, has numerous roles in innate and adaptive immune responses and inflammation. In the mature gut, pathogen-activated molecular patterns (PAMP)-specific region of a gut pathogen binds to its corresponding Toll-like receptor (TLR) on an enterocyte (EC) and causes release of NF-κB (38). The reaction enters the nucleus where genes mediating inflammation are turned on (38–40). In response to bacterial lipopolysaccharide (LPS), fetal ECs upregulate the NF-κB pathway and produce more of the chemokines, CXCL2 and CXCL8 (41).

### Tolerance and commensal bacteria

First exposure of the neonatal gut to LPS and antigens from non-pathogenic commensals may result in significant inflammatory responses. The mechanisms by which tolerance to non-pathogenic commensals is established at this most critical period require further understanding. Part of the process includes inhibition of the NF-κB pathway by commensal bacteria (40, 42).

As ECs mature, expression of surface TLRs reduces, as do downstream signaling complexes, while inhibitory factor kappa (iκB), a negative regulator, increases. This appears to provide “protection” without adverse inflammation during colonization (43). Alterations in innate immune response genes in fetal ECs contribute to NEC (44) and suggest that an inappropriate inflammatory response takes place in premature babies. Commensal bacteria exert a variety of effects on cytokine responses. These include production of pro-inflammatory cytokines via the IL-25–IL-23–IL-17 axis (45), possible downregulation of IL-17 in Th-17 cells resulting in diminished inflammation, and upregulation of IL-25. IL-25 is thought to suppress IL-23 by gut dendritic cells (DCs) with subsequent diminished IL-17 and an anti-inflammatory effect (45). Commensal bacteria also affect responses of CD103+ DCs to products of ECs (such as retinoic acid, TDF-β, and cytokine thymic stromal lymphopoietin (TSLP) that effectively dampen the adaptive immune response in those (46). In addition, commensal bacteria (such as *Bifidobacterium* sp.) may upregulate IL-10 production by DCs (47).

### Pathogen recognition

Pathogen recognition by innate immune cells initiates an immune response to infection. Key PAMPs instigate effector responses through activation of pathogen pattern recognition receptors (PRRs), which include well-characterized TLRs. Adults and children carry an increased infection risk if they carry TLR polymorphisms. The same is true for SIDS (48). Neonatal infection with Gram-positive and Gram-negative bacterial infections is associated with enhanced expression of TLR2 and TLR4 (38). Dysregulation of TLR4 expression is associated with development of NEC (49), commonly associated with neonatal sepsis. In addition to activation by exogenous PAMPs such as LPS or viral ssDNA, TLRs occurs through endogenous damage- or danger-associated molecular patterns (DAMPs), intracellular proteins, and inflammatory mediators released by damaged or apoptotic cells. These include endogenous Alarmin High-Mobility Group Box 1 (HMGB1), heat shock proteins, and uric acid; each contributes to the pathophysiology of septic shock. Dysregulated HMGB1 expression associated with progression of sepsis to septic shock (43, 50) perpetuates the inflammatory response. Disruption of EC tight junctions also occurs and leads to increased bacterial translocation (51) and bacteremia.

Recognition of PAMPS or DAMPS by PRRs results in activation of NFkB resulting in pro-inflammatory cytokine and chemokine production and induction of the IRF transcription factors that mediate production of type I interferon. The NOD-like receptor (NLR) family of proteins shares a number of common domains and many are involved in PAMP and DAMP sensing. The process results in NFkB activation and inflammatory gene expression (52, 53). NLRP proteins oligomerize directly or indirectly with caspase1 through the caspase recruitment domain (CARD) to form an inflammasome. Inflammasomes are essentially caspase-activating complexes.

The anti-inflammatory nature of IL-10 is well known, except, perhaps in SIDS where the cytokine might contribute to a fatally defective pathogen recognition (48). In the neonatal gut, IL-10 is reported to reduce inflammation as evidenced by inhibition of key parts of the unfolded protein response (UPR) (38, 54). Paneth and goblet cells respond to abnormal protein handling with the UPR, which results in local tissue inflammation through activation of immune cells including neutrophils (55).

### Role of innate lymphoid cells

These cells do not possess a specific antigen receptor; however, innate lymphoid cells (ILCs) are able to secrete a number of cytokines equivalent to those produced by the subsets (subsets T_H_2, T_H_17, and T_H_22.) of T helper cells. Subsets of ILCs have been demonstrated. ILC1 cells differ from natural killer (NK) cells in that they lack CD56, CD16, and CD94 NK cell markers as well as perforin, and granzyme B. ILCs function in lymphoid organogenesis, tissue remodeling, antimicrobial immunity, and inflammation, particularly at barrier surfaces (56). Their ability to respond promptly to insults inflicted by stress-causing microbes strongly suggests that ILCs are critical in first-line immunological defenses. A number of families of ILCs have been described. These include Rorγt-expressing cells involved in lymphoid tissue formation, mucosal immunity, and inflammation. Type 2 ILCs are important for helminth immunity, type 3 for mucosal integrity and healing.

Gut homeostasis depends on minimizing responses to commensal bacteria, which can lead to inflammatory bowel disease (IBD) in genetically predisposed individuals; but there is a need to retain the ability to recognize and control the growth of infectious pathogens (56). Group 3 innate lymphoid cells (ILC3) help maintain intestinal homeostasis by producing the cytokine IL-22, which promotes mucosal healing and maintains barrier integrity. Microbial signals trigger production of IL-23 and IL-1β; these stimulate ILC3s to produce IL-22, leading to induction of antibacterial peptides and epithelial cell regeneration (57). The cell type producing IL-23 in response to microbial signals is unclear and much debated; resident mononuclear phagocytes (MNPs), inflammatory monocytes, and conventional DCs have all been implicated. Longman et al. (57) provide a clue. In mice with *Clostridium rodentium* infection and in patients with colitis, CX_3_CR1^+^ MNPs are superior producers of IL-23 and IL-1β, and they are very efficient in inducing IL-22 production by ILC3 (56, 58).

The development of Peyer’s patches and ILC2 and ILC3 subsets depends on *Nfil3 (nuclear factor, interleukin 3-regulated)*. Loss of *Nfil3* selectively decreases Peyer’s patch formation and is associated with defective recruitment and distribution of ILCs within the patches. ILC subsets strongly express *Nfil3*. Deletion of *Nfil3 genes* adversely affects development of all subsets, so that *Nfil3^-/-^* knockout mice show increased susceptibility to infection or pro-inflammatory agents confirming the importance of the role of *Nfil3* in development of ILC subsets upon which the gut depends for protective immunity (59), especially against intestinal pathogens (60). Additionally, development of all innate lymphoid cell subsets depends on Nfil3.

## Gut Microbiome, Inflammation, and SIDS

The microbiome contributes to development and sustenance of the immune system. This includes protective immune effector function in the healthy host as well as in cases of disease. It is noted that SIDS cases frequently have been unwell in the days leading up to the death. Diarrheal symptoms are often reported (61). Bacteremia associated with viral gastroenteritis often with an accompanying fever is a known complication (62). Bacteremia without localizing source is also well known (63), and occult bacteremia is not uncommon in infancy (64). Such episodes of bacteremia are usually benign, indicating that most babies’ immune responses are able to cope without lethal consequences. By contrast, SIDS babies might have dysregulated responses (48). In these circumstances, it could be suggested that bacteremia could result in an overwhelming cytokine storm with consequent sepsis/toxemia resulting in the baby’s demise.

In addition to the pathogenic *E. coli* groups (Bettelheim and Goldwater, this issue), two groups of the gut microbiome have been investigated in relation to SIDS – *Bacteroides thetaiotaomicron* and *Clostridium* species.

### Gram-negative bacteria and inflammation

It is known that the NLRC4 inflammasome is activated through caspase1 by Gram-negative bacteria containing type III or type IV secretion systems, e.g., *Salmonella*, *Shigella*, *Legionella*, *Pseudomonas*, *Yersinia*, and some *E. coli*. NLRC4 specifically recognizes flagellin; consequently, non-motile strains are unable to activate caspase1. Flagellin protein alone has been shown to activate caspase1. Caspase1 activation appears to be dependent on a rod protein in the type III secretion system that contains a flagellin-like motif (52, 53).

Translocation of bacteria through an inflamed gut wall leading to bacteremia sets the stage for further induction of pro-inflammatory cytokines and perturbation of the clotting cascades. As IL-6 is elevated in the CSF of SIDS cases (65), it is reasonable to surmise that pro-inflammatory cytokines might be responsible for observed organ changes: brain microglial response (10); increased brain weight; myocardial acute inflammatory reaction (66). Pro-inflammatory cytokines are responsible for perturbation of the clotting cascade and loss of endothelial integrity (67) resulting in raised fibrin degradation products and the vasculopathy evidenced as intrathoracic petechial hemorrhages. SIDS babies frequently carry a low-producer polymorphism for the anti-inflammatory cytokine IL-10 (68), which could contribute to reduced control of pro-inflammatory cytokine release.

### *B*. *thetaiotaomicron*

*B. thetaiotaomicron*, a Gram-negative obligate anaerobe, colonizing the human gut, is a major endosymbiont of the human gut. It can hydrolyze and utilize as an energy source non-digestible polysaccharides and maltooligosaccharides (69). It contributes to bacteria–host symbiosis, postnatal intestinal development, physiology and metabolism of the host (70), and development of the immune system (71).

*Bacteroides thetaiotaomicron* is recognized as a major player in the adult intestinal microbiome and is useful as a model for the investigation of human–bacterial interactions. It degrades plant polysaccharides. In the infant, it is important during transition from breast milk to a high plant starch diet. It has been shown to stimulate intestinal angiogenesis in response to microbial products reaching the Paneth cells (72). Postnatally, *B. thetaiotaomicron* mediates formation of the mucosal gut barrier assisting in protection against pathogenic invasion seemingly through its effect on expression of species-specific protein antibiotics (73). *B. thetaiotaomicron* allows for adaptive carbohydrate foraging through its environmental sensing system (74). This results in stabilized food webs, and sustenance of bacterial communities (73). This could be important in protection against bacteria and/or toxins purportedly involved in SIDS pathogenesis. The species has been identified among a higher proportion of SIDS infants (30%) compared with control healthy babies (8.8%) (17).

### *Clostridium* spp

Lecithinase-positive clostridia and other clostridia are found significantly more often in formula-fed babies than breast-fed babies (75). Our study found SIDS babies had significantly higher rates of colonization with these anaerobes than live comparison babies (17). Formula feeding seemed to show a trend toward higher colonization rates with clostridia than breastfeeding (17). The role of clostridia in gut inflammation is discussed below. In the context of inflammation, the review by Schuijt et al. (76) considered the hypothesis that the gut has an important detrimental role in promoting systemic inflammation and infection in the critically ill. They demonstrated that during stress and mucosal hypoxia, the mucosa is damaged and host defenses break down allowing bacteria and toxin translocation thought to produce overwhelming inflammation, sepsis, and multiorgan failure (77, 78).

## Evidence of Infection in SIDS

Babies dying of SIDS are reported to have sweat-soaked clothing and bedding indicating a febrile episode in the last sleep (61). Rectal temperatures at autopsy of SIDS babies are elevated providing further evidence of fever during the last sleep (79). The basis for a link between the gut microbiome and SIDS is founded upon a substantial body of published evidence and congruence with factors associated with sepsis (Table 1). This provides an explanatory scheme for underlying pathogenic mechanisms involved in events leading to SIDS.

### Pathological findings

Consistent pathological findings include intrathoracic petechiae; liquid heart blood; heavy wet lungs; large heavy brain with microglial response (10); acute myocardial inflammatory reaction (66); elevated fibrin degradation products (80); and recent viral infection (61). These suggest a single, common patho-mechanism (5, 6). The finding of *S. aureus* and *E. coli* and other coliforms in normally sterile sites (66, 81, 82) supports the idea of bacteremia occurring as a plausible near-terminal event. Morris’ Common Bacterial Toxin Hypothesis (1, 2, 83) first indicated a key role of bacteremia in the final pathway. How the purported bacteremia arises seems to implicate gut integrity/permeability/immunity, which depend on the gut microbiome (84). Potentially, pathogenic *Clostridia* species are over-represented in SIDS babies (17); these could influence the integrity of the gut wall through either induction of an inflammatory response or mechanism involving disruption of EC tight junctions leading to increased bacterial translocation (51).

### Inflammation and pro-inflammatory cytokines

Inflammasomes are thought to be key in induction of inflammation through production of pro-inflammatory cytokines. Secretion of IL-1β mediates recruitment of cells to the site of proposed clostridia-mediated insult and could play a role in SIDS (52, 53). While gut inflammation is considered to be primary in the events leading to SIDS, the other side of the equation is defective responses to infection, e.g., a defective pathogen recognition pathway.

### Clinico-physiological events in SIDS

The clinical/physiological events observed in SIDS cases captured on memory monitors (85) can be explained by development of septic/toxic shock resulting from the release of pro-inflammatory cytokines secondary to a bacteremic episode or toxemia. These include fever, tachycardia followed by profound bradycardia, hypoxemia, and gasping (after asystole) (85).

### Evidence of bacteremia/toxemia in SIDS infants

The relatively low rate of sterile site infection (16–19% of SIDS cases) could argue against the bacteremia hypothesis. The finding of viable bacteria in a normally sterile site depends on the bacteria’s cultivability. Most of the gut microbiota are unlikely to grow on standard diagnostic artificial culture media (86), and this could contribute to low-positive culture rates. It remains highly probable that non-cultivable bacteria entering the blood stream could induce the cytokine storm thought to underlie the final events in SIDS.

### Brain and heart abnormalities in SIDS

The findings of brain abnormality (87) and of smaller than normal heart and kidneys (88) seem to relate to abnormal prenatal development. This was refuted by Guntheroth and Spiers (89) and needs to be revisited. The smaller than normal heart (with accompanying possible predisposition to arrythmia) could make the infant more vulnerable to profound immuno-inflammatory events associated with bacteremia/sepsis.

### SIDS risk factors

Risk factors for SIDS can be categorized into genetic, prenatal, and postnatal. These have been described previously (5, 6). Why prone sleep position is a risk factor has been explained by the acquisition of bacteria from contaminated surfaces (sofas, parental bed, being especially contaminated and high risk) (17). It is important to note that data purportedly showing a relationship between bedsharing and SIDS and inferring overlaying/asphyxia as cause (90) needs re-examination because the reason for bedsharing remains poorly described in published studies. It could be interpreted that bedsharing on the night of the last sleep came about because the baby was unsettled or unwell, indicating subtle symptoms of infection. This is supported in a systematic review of bed sharing in which illness and crying were frequently reported (91). The role of alcohol and drugs by co-sleeping parent(s) has also been interpreted as leading to overlaying. The alternative suggestion of lessened awareness in the co-sleeping parent might lead to failure to notice subtle symptoms of illness in the baby. Using overlaying with consequent asphyxiation as the major explanation of the risk factor of bedsharing requires re-evaluation. Mainstream researchers attribute a “respiratory” cause for intrathoracic petechiae. Differentiation between the intrathoracic petechiae observed in asphyxia and SIDS have been well described (92, 93) and show clear differences; yet, these findings are largely ignored by mainstream researchers.

## Future Research

Delineation of the gut microbiome in SIDS and healthy babies might provide additional clues to the processes underlying the role of the gut in SUDI. Extended immunohistological examination of the gut wall for evidence of ILCs and subtle gut inflammatory changes in SIDS babies might provide evidence of inflammation contributing to a functional failure of the gut wall to prevent bacterial translocation from the gut lumen into the bloodstream. Nfil3 gene polymorphisms should be sought as these could contribute to gut vulnerability. In the context of bacteremia, efforts using PCR technology should be made to exclude/demonstrate the presence of non-cultivable bacteria in normally sterile sites. The gut microbiome observed in SIDS might correlate with the presence of ILCs and a “pro-inflammatory gut microbiome.” Such findings might contribute to a new definition of SIDS. The corollary of establishing a possible “anti-inflammatory” gut microbiome in healthy babies via diet or other means (pre- or probiotic) could be explored as a “natural” mechanism of protecting babies in the future but not forgetting the messages advocating a safe sleeping environment and parental avoidance of cigarette smoke and other harmful drugs.

## Conflict of Interest Statement

The author declares that the research was conducted in the absence of any commercial or financial relationships that could be construed as a potential conflict of interest.
